# The relationship between smartphone addiction and symptoms of depression, anxiety, and attention-deficit/hyperactivity in South Korean adolescents

**DOI:** 10.1186/s12991-019-0224-8

**Published:** 2019-03-09

**Authors:** Seung-Gon Kim, Jong Park, Hun-Tae Kim, Zihang Pan, Yena Lee, Roger S. McIntyre

**Affiliations:** 1 Department of Psychiatry, School of Medicine, College of Medicine, Chosun University, 365 Pilmundaero, Dong-gu, Gwangju, 501-717 Republic of Korea; 2 Department of Preventive Medicine, College of Medicine, Chosun University, Gwangju, Republic of Korea; 3 Department of Psychiatry, University of Calgary, Calgary, AB Canada; 4 Mood Disorders Psychopharmacology Unit, University Health Network, Toronto, ON Canada; 5 Institute of Medical Science, University of Toronto, Toronto, ON Canada; 6 Departments of Psychiatry and Pharmacology, University of Toronto, Toronto, ON Canada

## Abstract

**Background:**

Excessive smartphone use has been associated with numerous psychiatric disorders. This study aimed to investigate the prevalence of smartphone addiction and its association with depression, anxiety, and attention-deficit hyperactivity disorder (ADHD) symptoms in a large sample of Korean adolescents.

**Methods:**

A total of 4512 (2034 males and 2478 females) middle- and high-school students in South Korea were included in this study. Subjects were asked to complete a self-reported questionnaire, including measures of the Korean Smartphone Addiction Scale (SAS), Beck Depression Inventory (BDI), Beck Anxiety Inventory (BAI), and Conners-Wells’ Adolescent Self-Report Scale (CASS). Smartphone addiction and non-addiction groups were defined using SAS score of 42 as a cut-off. The data were analyzed using multivariate logistic regression analyses.

**Results:**

338 subjects (7.5%) were categorized to the addiction group. Total SAS score was positively correlated with total CASS score, BDI score, BAI score, female sex, smoking, and alcohol use. Using multivariate logistic regression analyses, the odds ratio of ADHD group compared to the non-ADHD group for smartphone addiction was 6.43, the highest among all variables (95% CI 4.60–9.00).

**Conclusions:**

Our findings indicate that ADHD may be a significant risk factor for developing smartphone addiction. The neurobiological substrates subserving smartphone addiction may provide insights on to both shared and discrete mechanisms with other brain-based disorders.

## Introduction

As a novel technology combining internet and communication, smartphones have become an essential part of daily life. The number of smartphone users has been rapidly increasing globally, with Asia and Europe showing the largest increases. For example, there are 2.21 smartphones for every individual in Hong Kong, and the number of active smartphone lines in Switzerland exceeds its population [1]. In South Korea, according to reports from 2014, 78.6% of the population possessed a smartphone device despite South Korea’s short history of smartphone dissemination beginning with the iPhone in 2009 [2].

The use of smartphones facilitates access to disparate social media opportunities, messaging capabilities, Internet access, as well as other purposes. Moreover, smartphone is rapidly replacing personal computer as the preferred mode of engaging online activity. However, excessive smartphone use may have negative effects on adolescents, especially since adolescence is a sensitive period characterized by the occurrence of many changes physiologically, psychologically and socially. Cell-phone use has been noted to decline with age, with individuals aged 14–20 spending the most time using their cell phones [3, 4]. This suggests that this age group can be particularly vulnerable to the negative effects of cell-phone use due to risks of childhood onset mental disorders characterized by a disturbance in impulse control and cognitive flexibility. As such, it is particularly important that the effect of smartphone addiction during adolescence should be investigated to implement effective prevention  and management plans.

Despite the lack of discrete diagnostic criteria for smartphone addiction, the definition of addiction in general has been expanded to include areas of behavioral addiction (i.e., gambling, internet gaming) [5]. Most smartphone users show high degree of Internet use and there may exist further association with Internet gaming addiction [6]. Pathological gambling under “Impulse Control Disorders” in the Diagnostic and Statistical Manual of Mental Disorders 4th Edition [7] was replaced by gambling disorder, classified under “Substance-Related and Addictive Disorders”, and Internet gaming disorder was added to “Conditions for Further Study” in the DSM-5 [8]. These changes highlight the growing importance of understanding behavioral addictions and demonstrate the need for research to understand rapidly increasing rates of smartphone addiction.

Numerous psychiatric problems related to excessive smartphone use have been identified, including depressive symptoms [9], anxiety [9], and low self-esteem [10]. One study has reported greater severity of smartphone addiction with higher expectations and demands from parents or teachers, higher levels of stress, and higher severity of depressive symptoms [11]. However, in a study of 755 university students, smartphone addiction was found to be significantly associated with depression, anxiety, obsessive–compulsive symptoms, and impulsivity [12]. In addition, trait impulsivity has been implicated in behavioral addiction [13]. Indeed, higher impulsivity was noted in the risky smartphone use group compared to the normal use group in a recent correlational study conducted in Korean university students [14].

Given their relatively underdeveloped impulse and self-control, as well as their increasing use of smartphones, psychiatric and psychological difficulties in adolescents related to smartphone use are highly likely. Although the definition of smartphone addiction has not been well established in the literature thus far, impulsivity, one of the hallmark symptoms of ADHD, is noted to have important implications in behavioral addictions [15]. Despite the growing interest and large number of new studies around smartphone addiction, no large-scale epidemiological studies have been conducted on the relationship between smartphone addiction and ADHD in adolescents. Indeed, while there have been studies investigating the association between ADHD symptoms and screen time [16] as well as internet addiction [17], they are largely limited to adult population and not specific to the entity of smartphone addiction. As such, this study aimed to investigate the prevalence of smartphone addiction and its association with depression, anxiety, and ADHD symptoms in a large sample of Korean adolescents.

## Methods

### Study settings and population

A sample of 5051 students was recruited from 155 middle and high schools in Gwangju, South Korea. Gwangju, with a population of 1.5 million people, is one of the 10 metropolitan cities in South Korea. The survey was conducted in December 2013 across the five districts of the city. The target population of this study consisted of grade 7–12 students attending middle or high school in the region. Every school was assigned a specific grade from which one class would be randomly selected to participate in the survey. Before data collection, we requested cooperation from the educational office of Gwangju Metropolitan City who promised full cooperation. Trained researchers visited classrooms, explained the procedures, and administered a self-report questionnaire to students. Students were assured of the voluntary, completely anonymous, and confidential nature of the survey. All students agreed to participate in the study, and we obtained a written consent from them. The questionnaire developed for this study was written in Korean and took about 30 min to complete.

### Measures

Demographic information included questions about sex, age, grade year, socio-economic status, and family structure. Sex was analyzed as a dichotomous variable (male = 0, female = 1) and age was assessed in years. Students were asked to identify their own perceived socioeconomic status, divided into high, middle, and low. We were unable to obtain an objective measure of socioeconomic status such as household income given the anonymous nature of our study design. Family structure was divided into three types of households: two (both) parents, single parent, and other.

To measure the severity of smartphone addiction, 15 items derived from the Korean Smart Addiction Scale (SAS) for Youth were used [18]. This scale was developed by the National Information Society Agency, sponsored by the South Korean government, and by drawing on existing measurement tools of Internet addiction. The SAS contains four subdomains: (1) disturbance of adaptive functions, (2) virtual life orientation, (3) withdrawal, and (4) tolerance. These items are scored on a 4-point Likert scale, ranging from never (1) to always (4). Total score of SAS ranges from 15 to 60 points. High-risk group is defined as total score greater than 45, and the potential risk group is defined as total score of 42 to 44. In this study, high risk and potential risk groups were defined as the addiction group, drawing upon previous research using the same tool [19] in South Korea. The Cronbach’s alpha in this study was 0.864.

To measure the degree of depression, we used the Beck Depression Inventory (BDI) composed of 21 items [20]. Each question inquires about the respondents’ particular symptoms and changes in mood in the past week on a 4-point scale (from 0 to 3). The validity and reliability of the BDI in a Korean population was previously established and suggested a cut-off point of 16 for depression in grade 7 students or older (above 12 years) [21]. As such, those who had a score of 16 and above were included in the depression group. The Cronbach’s alpha in this study was 0.894.

Regarding the anxiety symptoms, we used the Beck Anxiety Inventory (BAI) [22]. The scale consists of 21 items rated on a 4-point scale from zero to three. The tool has been validated in a Korean population as well [23]. We determined anxiety group using points of 16 and above, consistent with moderate level of anxiety in the scale and the suggested cut-off for clinical purposes [24]. This cut-off was further supported by a recent psychometric meta-analysis of BAI by Bardhosi et al. [25]. The Cronbach’s alpha in this study was 0.895.

To assess the symptoms of attention-deficit hyperactivity disorder (ADHD), we used the Conners-Wells’ Adolescent Self-Report Scale (CASS) [26]. The scale consists of 27 items rated on a 4-point scale (from 0 to 3). Its validity and reliability in Korean population were previously established [27]. The cut-offs in the Korean version are as follows: 41 points from 7th to 8th grade, 44 points in 9th grade, and 42 points from 10th to 12th grade. Respondents were divided into ADHD group and Non-ADHD group in accordance with the above cut-offs. In this study, the Cronbach’s alpha was 0.896.

In addition, we assessed smoking and alcohol use history using a single question for each: “Have you had at least a single alcoholic drink in the past month?” and “Have you smoked at least one cigarette in the past month?” The answer was recorded as a dichotomous variable (No = 0, Yes = 1).

### Statistical analysis

SPSS for Windows (ver. 21.0; SPSS Inc., Chicago, IL, USA) was used for statistical analysis. Participants were assigned to addiction group or normal group using the aforementioned definitions. Demographic characteristics were reported as frequency and percentage. Chi-square test was used to compare addiction and normal groups on characteristics of sex, socio-economic status, family structure, depression, anxiety, ADHD, smoking, and alcohol use. Pearson correlation analysis was performed to determine the correlation between smartphone addiction scores and other variables of interest. Finally, multivariate binary logistic regression analysis was performed to assess the influence of gender, depression, anxiety, ADHD, smoking, and alcohol use on smartphone addiction. The analysis was completed using backward method, with addiction group and normal group as dependent variables and female sex, depression group, anxiety group, ADHD group, smoking group, and alcohol groups as independent variables. A *p* value of less than 0.05 was considered to indicate statistical significance.

## Results

Among the 5051 students recruited for the study, 539 were excluded due to incomplete responses. Thus, a total of 4512 students (45.1% male, *n* = 2034; 54.9% female, *n* = 2478) were included in this study. The mean age of the subjects was 15.15 (SD = 1.62). The sociodemographic characteristics of the subjects are summarized in Table 1. For reference, 4060 students (87.8%) were smartphone owners (84.2% of male, *n* = 1718 of 2041; 90.6% of female, *n* = 2342 of 2584) among the 4625 students who responded to the question of smartphone ownership (426 did not respond).

**Table 1:** Demographic characteristics of the subjects (*n* = 4512)

	*n*	%
Age (mean ± SD)	15.15 ± 1.62	
Sex		
Male	2034	45.1
Female	2478	54.9
Grade year		
7th	891	19.7
8th	994	22
9th	689	15.3
10th	749	16.6
11th	776	17.2
12th	413	9.2
SES		
High	1219	27
Middle	2551	56.5
Low	742	16.4
Family structure		
Both parents	3939	87.3
Single parent	491	10.9
Other	82	1.8

*SD* standard deviation, *SES* socio-economic status

Table 2 shows clinical characteristics between smartphone addiction and normal groups. Of the 4512 participants, 338 (7.5%) were categorized to the addiction group, while 4174 belonged to the normal group. The mean age in the addiction group and normal group was 15.15 ± 1.63 and 15.09 ± 1.44, respectively, with no statistical difference between the groups (*t* = 0.744, *p* = 0.458). Furthermore, socio-economic status and family structure had no statistical difference between the groups (*χ*^2^ = 3.912, *p* = 0.141; *χ*^2^ = 0.685, *p* = 0.710). Apart from age, socio-economic status, and family structure, all other variables showed statistically significant differences between the addiction group and the normal group. These include: female sex (OR 1.75, 95% CI 1.38–2.21), depression (OR 4.15, 95% CI 3.26–5.28), anxiety (OR 4.41, 95% CI 3.43–5.64), cigarette smoking (OR 2.06, 95% CI 1.44–2.96), and alcohol use (OR 1.62, 95% CI 1.22–2.16). The largest difference among all variables was noted with ADHD symptoms. Compared to 26.0% of addiction group also belonging to the ADHD group, only 3.4% in the normal group were in the ADHD group. The odds ratio for smartphone addiction in ADHD group compared to non-ADHD was 10.14 (*χ*^2^ = 335.003, *p* < 0.001).

**Table 2:** Comparison of clinical characteristics between the addiction and normal group

Variables	Total (*n* = 4512)	Addiction group (*n* = 338)	Normal group (*n* = 4174)	*χ* ^2^	OR	95% CI
	*n* (%)	*n* (%)	*n* (%)			
Sex				22.108***	1.75	1.38–2.21
Male	2034 (45.1)	111 (32.8)	1923 (46.1)			
Female	2478 (54.9)	227 (67.2)	2251 (53.9)			
SES				3.912		
High	1219 (27.0)	83 (24.6)	1136 (27.2)			
Middle	2551 (56.5)	187 (55.3)	2364 (56.6)			
Low	742 (16.5)	68 (20.1)	674 (16.1)			
Family structure				0.685		
Both parents	3939 (87.3)	292 (86.4)	3647 (87.4)			
Single parent	491 (10.9)	38 (11.2)	453 (10.9)			
Other	82 (1.8)	8 (2.4)	74 (1.8)			
Depression				153.003***	4.15	3.26–5.28
Yes	622 (13.8)	122 (36.1)	500 (12.0)			
No	3890 (86.2)	216 (63.9)	3674 (88.0)			
Anxiety				157.998***	4.41	3.43–5.64
Yes	528 (11.7)	111 (32.8)	417 (10.0)			
No	3984 (88.3)	227 (67.2)	3757 (90.0)			
ADHD				335.269***	10.14	7.54–13.63
Yes	228 (5.1)	88 (26.0)	140 (3.4)			
No	4284 (94.9)	250 (74.0)	4034 (96.6)			
Smoking				16.12***	2.06	1.44–2.96
Yes	279 (6.2)	38 (11.2)	241 (5.8)			
No	4233 (93.8)	300 (88.8)	3933 (94.2)			
Alcohol				11.354**	1.62	1.22–2.16
Yes	598 (13.3)	65 (19.2)	533 (12.8)			
No	3914 (86.7)	273 (80.8)	3641 (87.2)			

*SES* socio-economic status, *ADHD* Attention-Deficit Hyperactivity Disorder, *OR* odds ratio, *CI* confidence interval

** *p* < 0.01, *** *p* < 0.001

Table 3 shows the Pearson correlation coefficients of smartphone addiction with other variables. Total smartphone addiction score showed greatest correlation with total CASS score (*r* = 0.427, *p* < 0.001). The total SAS score was also associated with total BDI score, total BAI score, female sex, smoking group, and alcohol use group in a statistically significant manner.

**Table 3:** Pearson’s correlations of variables

	1	2	3	4	5	6	7
1. Sex	1						
2. SAS	0.127**	1					
3. BDI	0.145**	0.327**	1				
4. BAI	0.100**	0.320**	0.654**	1			
5. CASS	− 0.037**	0.427**	0.539**	0.559**	1		
6. Smoking group	− 0.186**	0.090**	0.071**	0.087**	0.142**	1	
7. Alcohol group	− 0.124**	0.078**	0.092**	0.115**	0.137**	0.407**	1

Sex, smoking group and alcohol group was used as dummy variable (female = 1, smoking group = 1, alcohol group = 1)

*SAS* smartphone addiction scores, *BDI* Beck Depression Inventory, *BAI* Beck Anxiety Inventory, *CASS* Conners-Wells’ Adolescent Self-Report Scale

** *p* < 0.01

To identify the variables associated with smartphone addiction, multivariate logistic regression analyses were performed. All variables showing statistically significant difference between addiction group and normal group were entered and analyzed using backward method. In the goodness-of-fit test of the regression analysis model, the − 2 log likelihood was 2110.304 and statistically significant (*p* < 0.001). In the first model tested, alcohol use had no statistically significant effect on smartphone addiction (*B* = 0.161, OR = 1.174 *p* = 0.375, 95% CI 0.823–1.675) and was, thus, removed from the final model. Table 4 shows the final model of the analysis; the odds ratio for smartphone addiction of female sex to males was 2.01 (95% CI 1.54–2.61). Odds ratio of ADHD group compared to non-ADHD group for smartphone addiction was 6.43, highest among all variables (95% CI 4.60–9.00).

**Table 4:** Multivariate binary logistic regression

	*B*	SE	Wald	OR	95% CI
Female	0.700	0.134	27.320	2.01***	1.54–2.61
Depression group	0.644	0.154	17.544	1.90***	1.40–2.57
Anxiety group	0.628	0.161	15.213	1.87***	1.36–2.56
ADHD group	1.862	0.171	118.199	6.43***	4.60–9.00
Smoking group	0.717	0.21	11.705	2.04**	1.35–3.09

All variables was used as dummy variable (female = 1, depression group = 1, anxiety group = 1, ADHD group = 1, smoking group = 1)

*ADHD* Attention-Deficit Hyperactivity Disorder, *OR* odds ratio, *CI* confidence interval

** *p* < 0.01, *** *p* < 0.001

## Discussion

This study investigated the effects of depression, anxiety, and ADHD symptoms on smartphone addiction among adolescents. The overall prevalence of smartphone addiction was 7.5% in our sample, with higher rate of addiction found among females compared to males. Those in the smartphone addiction group were more likely to have significant symptoms of depression, anxiety, ADHD, and to use tobacco and alcohol compared to those in the normal group. All of the above factors were found to be correlated with smartphone addiction. Multivariate logistic regression showed female sex, depression, anxiety, ADHD, and smoking to have statistically significant effects on smartphone addiction. To our knowledge, this is the first large-scale epidemiological study investigating the relationship between ADHD symptoms and smartphone addiction in the adolescent population.

The prevalence of smartphone addiction in our study sample of adolescents was found to be 7.5%. According to the previous studies in Korean population using SAS (used in our study), the prevalence of smartphone addiction was 4.9% among 599 middle school students (7th–9th grade, 392 male and 207 female) [19] and 10.6% among 322 university students (212 male, 108 female) [14]. In Europe, one study targeting 1519 young people (15–21 years or older) found prevalence of smartphone addiction to be 16.9% [28]. Although it is difficult to interpret this variation given the paucity of existing literature investigating prevalence of smartphone addiction in different regions, a meta-analysis of internet addiction also found significant variation in prevalence ranging from 0.6% in Italy to 26.7% in Hong Kong using numerous assessment tools and cut-off scores [29]. Thus, the variation in the current literature regarding the prevalence of smartphone addiction may be due to inconsistent study methods, study sample, and geographical variance. Further research is necessary using a unified assessment tool and taking such regional variations into account.

The rate of smartphone addiction was higher in females compared to males, consistent with previous reports [3, 29]. This may be related to the more relationship-oriented nature of females and differences in how the smartphone is used. Previous research suggests that men primarily use the smartphone for work-related purposes, internet search, and entertainment, while women spend more time using social network services or instant messaging, resulting in more frequent use of smartphones [30]. Thus, men tend to spend more time on work-related activities, while women seek to enhance their social relationships, resulting in more time spent using smartphones. Considering the relative vulnerability of females to smartphone addiction, further investigation into patterns of smartphone use and related risk factors is warranted.

In addition, the presence of depression, anxiety, and ADHD symptoms significantly increased the odds of meeting criteria for smartphone addiction. This is in keeping with literature reporting depression as a risk factor for Internet and smartphone addiction [12]. It is also consistent with previous studies reporting a significant relationship between anxiety and smartphone addiction [31], as well as social anxiety disorder and Internet addiction [32]. A review of literature on problematic smartphone use highlighted both the direction and possible explanations of relationship between psychopathology and problematic smartphone use [33]. These include excessive reassurance seeking and avoidance behaviours with smartphones (psychopathology causing problematic smartphone use), sleep disturbance (problematic smartphone use causing psychopathology), and interaction between these factors to further drive each other in a bidirectional relationship.

Even after controlling for other variables, the presence of ADHD demonstrated strongest association with smartphone addiction. Those with ADHD tend to be impulsive, have difficulty controlling their behaviors, and are highly sensitive to reward. The feeling of being in control of a target, co-occurring stimuli, and freedom to express oneself associated with smartphone use can provide high degrees of motivation and reward for adolescents with ADHD. Preference for immediate reward with dislike for delay of reward was noted to be a characteristic of ADHD in the past [34]. Billieux et al. [35] suggested that elements of impulsivity, such as impatience, low perseverance, and length of cell-phone possession are strong predictors of addiction. Adolescents with ADHD can find smartphone use more attractive than those without, given the instant response and reward offered through smartphone use and its portability. Functional Magnetic Resonance Imaging (fMRI) studies have reported abnormal brain activity during behavioral inhibition in adolescents with ADHD [36]. The deficits in self-control and inhibition can lead to more prominent difficulties with excessive use of smartphones and worsening of ADHD symptoms. Given that Internet addiction is subserved by similar brain substrates as ADHD (e.g., executive cognitive control) [37], and given its similarity to smartphone addiction, it can be hypothesized that the neurobiological characteristics of adolescents with ADHD play a significant role in its relationship with smartphone addiction. This is further in keeping with a recent study by Kahraman and Demirci [38] which identified a significant association between Internet Addiction Scale scores and ADHD which persisted after controlling for effects of other variables including depression and anxiety scores.

Although smoking and alcohol use was associated with smartphone addiction in our study, the result of multivariate logistic regression analysis showed statistical significance only for cigarette smoking. Sanchez and Otero [39] found cell-phone abuse to be associated with academic failure, depressive symptoms, smoking, and other substance use disorders. Furthermore, Toda et al. [40] found associations between cell-phone use and smoking only in men, and no association was found with alcohol use. Addiction to substances such as alcohol and nicotine is related to impulse control difficulties and low self-esteem, and these factors can likewise play an important role in smartphone addiction. It can be hypothesized that those with vulnerability to addiction are more susceptible to developing other addictions, including that of smartphone and any other substances. Involvement in one health risk behavior may lead to further risky behaviors, resulting in complex health problems. More research is necessary to solidify our understanding of the relationship between smartphone addiction and other substance use, including alcohol and cigarettes, with preventative and management strategies to address this issue.

There are number of limitations to our current study. Firstly, this is a cross-sectional study, and as such, causality could not be established between smartphone addiction and depression, anxiety, ADHD, smoking, and alcohol use. Prospective studies to further establish interaction and causality between smartphone addiction and these variables are necessary. Secondly, because the study population was limited to those attending middle or high schools in a specific city, the sample may not be a true representation of other adolescents in South Korea. However, given the large number of students that were surveyed in schools across the city, it represents the characteristics of adolescents in the sampled region quite well. Given that the study was limited to adolescents, generalization to other age groups is difficult. Finally, the survey was based entirely on self-report, including collection of demographic variables. We were unable to obtain an objective measure of socioeconomic status such as household income or school performance given the anonymous nature of our study design. Future studies should seek to implement objective measures to further clarify the associations.

## Conclusion

Despite the aforementioned limitations, our study was able to identify an association between smartphone addiction and depression, anxiety, and ADHD in a large sample of adolescents. It is particularly important as research in smartphone addiction continues to be limited despite the prominence of ADHD in adolescence. Our study showed a significant association between symptoms of ADHD and smartphone addiction highlighting the need for further investigation and efforts towards prevention. Characteristics of smartphone addiction and its association with sex warrants further attention as females continue to be more susceptible to smartphone addiction, consistent with previous findings. Efforts should be made to understand the differences in smartphone addiction between adolescents and adults, as well as geographical and cross-cultural differences. Finally, longitudinal studies to investigate psychosocial factors and their influence on smartphone addiction, as well as those exploring genetic and physiological elements of smartphone addiction, are warranted.

## Abbreviations

ADHD: Attention-Deficit Hyperactivity Disorder
BDI: Beck Depression Inventory
BAI: Beck Anxiety Inventory
CASS: Conners-Wells’ Adolescent Self-Report Scale
SAS: Smartphone Addiction Scale
